# Splenic Abscess after Sleeve Gastrectomy

**DOI:** 10.1155/2020/4850675

**Published:** 2020-04-12

**Authors:** Rany Aoun, Michel Gabriel, Elias El Haddad, Roger Noun, Ghassan Chakhtoura

**Affiliations:** ^1^Department of Digestive Surgery, Hotel Dieu de France Hospital, University Saint Joseph Medical School, Beirut, Lebanon; ^2^Department of Radiology, Hotel Dieu de France Hospital, University Saint Joseph Medical School, Beirut, Lebanon

## Abstract

Splenic abscess is a very rare complication of laparoscopic sleeve gastrectomy (LSG). Clinical presentation includes fever, leucocystosis, and abdominal pain. CT SCAN is a must for diagnosis. The preferred treatment is either conservative, with intravenous antibiotics and percutaneous drainage, or splenectomy. We report the thirteen case of a splenic abscess after LSG. In our patient, the abscess occurred three weeks after LSG in a 21-year-old man, and it was successfully treated conservatively.

## 1. Introduction

Morbid obesity is nowadays a common disease affecting large amount of adults worldwide. Laparoscopic sleeve gastrectomy (LSG) is a simple procedure to treat morbid obesity. However, complications occur and include hemorrhage, leak of luminal contents, obstruction, and infection (wound and intra-abdominal abscess). We report the case of a patient who underwent LSG, complicated three weeks after surgery by a splenic abscess. And to the best of our knowledge, there were only twelve cases reported in the literature [1–8].

## 2. Case Presentation

A 21-year-old obese male with a body mass index of 45 kg/m^2^ underwent LSG in our department in April 2018. The patient did not have any past medical or surgical history. No systemic CT scan was done preoperatively, only gastroscopy that showed normal mucous of the stomach. The operation was very smooth without any complications: no splenic infarction nor a tear was detectable. No hemostatic agents were used. Immediate postoperative course was uneventful, and the patient was discharged on day 2 after tolerating a clear diet. No routine CT scan or opacification was made. Three weeks after surgery, the patient presented high fever and chills and took 24 hours of oral antibiotics before consulting his surgeon who decided to rehospitalize the patient.

**Figure 1:**
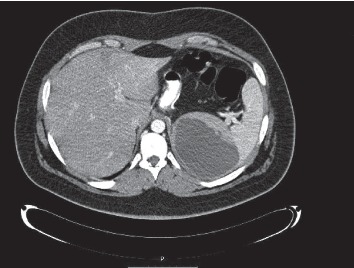
Splenic abscess of 10 cm with no evidence of leakage.

**Figure 2:**
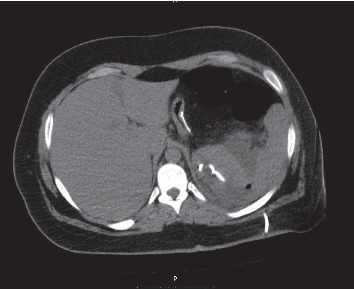
Percutaneous drainage of the splenic abscess.

At admission, his temperature was 38.5°C, and pulse rate was 120/min with a normal blood pressure. The blood results revealed 17,700 WBC and a CRP level at 295. Abdominal CT scan (Figure 1) showed a splenic abscess of 10 cm with no evidence of leakage. Initial management included hydration, intravenous antibiotics (piperacillin/tazobactam), and percutaneous drainage (Figure 2). The liquid culture was negative. The drain was removed after two days to avoid the risk of splenic hemorrhage without follow-up imaging.

**Figure 3:**
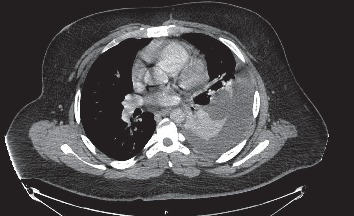
Pleural effusion and residual splenic abscess of 2 × 3 cm.

The patient got better clinically and a full fluids diet was initiated, but he continued to experience low grade fever with a WBC count of 14,400 and CRP at 240. A new abdominal CT scan showed a residual splenic collection of 2 × 3 cm with a pleural effusion (Figure 3). The pleural effusion was drained, and it revealed a transudate liquid with negative culture. The splenic collection was considered as a residual abscess, and then we decided to upgrade antibiotherapy to a larger broad-spectrum one (imipenem) without radiologic or surgical drainage of the residual splenic abscess. A total of ten days with intravenous antibiotic treatment was necessary before fever disappearance. The patient was discharged under IM ertapenem for 10 days.

Follow-up at 6 months, the patient was completely asymptomatic. Blood tests showed a normal count of WBC and CRP level.

## 3. Discussion

In general, splenic abscess can result from multiple causes such as neoplasia, immunodeficiency, trauma, splenic infarction, endocarditis, and sickle cell disease [9]. It was also reported after some gastric procedures like Nissen fundoplication and gastrectomy for cancer [10].

Splenic abscess after LSG is a very rare condition, and the literature enumerates twelve patients who experienced this condition (Table 1) [1–8]. The mechanism of formation of splenic abscess described by the previous authors include iatrogenic splenic injury during surgery, splenic ischemia after LSG, extension from a gastric staple-line leak, and temporary immune suppression in the immediate postoperative course.

In our case, there was no evidence of leakage nor a spleen ischemia or a spleen injury during the operation. Therefore, the formation of splenic abscess could be related to temporary immune suppression that results from rapid weight loss, limited oral intake, and a transient bacteraemia as it was noticed by Sakran et al. [2].

In most of the cases, the patient presented with fever, leucocytosis, and left upper quadrant pain. Our patient experienced the same symptoms. The diagnosis was made on CT scan of the abdomen, as it remains the gold standard for diagnosis of splenic abscess [9].

Splenic abscesses tend to be polymicrobial [11, 12], so they should be treated with broad-spectrum antibiotics [2, 4]. Enteric Gram negative and Gram positive were the common organisms as presented in Table 1. In our case, the culture was negative because the patient started on antibiotics without our knowledge before being admitted.

Solitary spleen abscesses are actually treated with percutaneous or laparoscopic drainage, in order to preserve the spleen. When symptoms persist or when multiple abscesses exist, splenectomy remains the definitive management [9, 11, 12]. However, our patient experienced a good evolution and a stable condition under conservative treatment, although a residual collection was documented on the last CT scan.

After all, would a splenectomy be beneficial even if the patient is completely asymptomatic, knowing that he is actually 6 months off treatment postoperatively?

## 4. Conclusion

Splenic abscess is a very rare complication after LSG. The etiology of formation of splenic abscess needs further studies. The patient must benefit from a conservative treatment based on antibiotics and percutaneous drainage. The decision to undergo a splenectomy will be based on clinical status and response to treatment.

## Figures and Tables

**Table 1:** Characteristics of the different cases.

Reference	Age/sex	Immunosuppression	Immediate complications	Post-op day of presentation	Treatment	Evidence of leakage	Culture
Rojas et al. [1]	46/F	No	Haemoperitoneum, splenic hilum, and hepatic injury	14	IV AB, percutaneous drainage	Yes	*S. anginosus*
Sakran et al. [2]	36/F	No	No	60	IV AB, splenectomy	—	*Streptococcus* spp, *E. coli*, *Enterococcus faecalis*
Sakran et al. [2]	35/F	—	No	75	IV AB, percutaneous and laparoscopic drainage	No	*Staphylococcus* spp, *Enterobacter cloacae*, *Streptopcoccus mitis* and *S. oralis*
Avulov et al. [8]	19/M	No	No	14	IV AB, percutaneous drainage, and splenectomy	No	*Salmonella* spp.
Schiavo et al. [3]	26/M	—	No	77	IV AB, percutaneous drainage	No	*S. anginosus*
Singh et al. [4]	44/M	No	No	70	IV AB, percutaneous drainage, and splenectomy	No	*Klebsiella pneumonia, Streptococcus pneumonia, Acinetobacter* spp.
Cervera-Hernandez and Pohl [5]	45/F	No	No	20	IV AB, percutaneous drainage	No	*S. anginosus*
Nassour et al. [6]	22/F	No	No	18 months	IV AB, splenectomy	Yes	*Streptococcus* spp. and *Fusobacterium*
Nassour et al. [6]	39/M	No	Superior splenic infarction	90	IV AB and oral AB, splenectomy	No	—
Nassour et al. [6]	68/F	Yes	Superior splenic infarction	12 months	IV AB and percutaneous drainage	No	—
Abdelhady et al. [7]	22/F	No	Partial splenic tear drainage	30	IV AB and percutaneous	No	Streptococci, *E. coli*
Abdelhady et al. [7]	26/M	No	No	21	IV AB, percutaneous drainage and splenectomy	Yes	*E. coli*
Our study	21/M	No	No	21	IV AB and percutaneous drainage	No	Negative
